# Microsurgical DREZ lesions for the control of cancer-related pain

**DOI:** 10.3171/2020.7.FOCVID2033

**Published:** 2020-10-01

**Authors:** Edoardo Mazzucchi, Andrei Brinzeu, Patrick Mertens, Marc Sindou

**Affiliations:** 1 Mater Olbia Hospital, Olbia, Italy;; 2 Université de Lyon, Université Claude Bernard Lyon 1, Lyon, France; and; 3 Universitatea de Medicina si Farmacie Victor Babes, Timisoara, Romania

**Keywords:** dorsal root entry zone, DREZ lesioning, Pancoast-Tobias tumor, tumor in the lumbosacral plexus, cancer pain

## Abstract

Pain in patients with cancer is a major problem, and sometimes it is necessary to surgically interrupt pain pathways to effectively control refractory pain. Surgical lesion of the dorsal root entry zone (DREZ) was first performed in 1972 for the treatment of pain related to a Pancoast-Tobias tumor. The rationale of DREZotomy is to preferentially interrupt the nociceptive inputs in the lateral part of the DREZ and the ventrolateral (excitatory) part of the dorsal horn. Microsurgical DREZotomy is one technique for DREZ lesioning that is suited for tailored control of pain in patients in good general condition who are experiencing pain in a well-defined territory.

The video can be found here: https://youtu.be/JtLQDP7gYSQ

## Transcript

This is a video on how to perform lesioning in the dorsal root entry zone, using a microsurgical method, for the control of pain in patients with cancer.

**0:27 Background.** Pain is a frequent and a notoriously difficult-to-treat problem in patients with cancer, and while many medical methods are available, sometimes it is necessary to surgically interrupt pain pathways in order to control the pain when it becomes refractory.^1,2^ Mesencephalic tractotomy, anterolateral cordotomy, and myelotomy (Supplemental Fig. 1) have all been used for various types of cancer.^2,3^ In this video, we demonstrate a technique that is particularly adapted for cancers with limited extension resulting in pain in a well-defined territory, and that is microsurgical DREZotomy at cervical and lumbar levels.

### Rationale

**1:05** Rationale of DREZ lesioning is based on microanatomical observations performed in Lyon (Supplemental Fig. 2) concerning the disposition of fibers within the dorsal root at the point of entry in the spinal cord according to their size and myelination type, and their projection on the dorsal horn.^4,5^ This can easily be observed in histological preparations (Supplemental Fig. 3) of the spinal cord evidencing both myelin and nonmyelin fibers. In the more peripheral portion of the root (Supplemental Fig. 4), fibers are disposed randomly regardless of their size and myelination. As they approach the spinal cord—at the transitional zone (Redlich-Obersteiner or pial ring of Tarloff)—the large-caliber myelinated fibers (carrying tactile and proprioceptive information) will tend to group toward the medial side of the root to reach the dorsal column. Conversely, smaller myelinated fibers and unmyelinated ones (carrying pain and temperature information) tend toward the ventrolateral part of the root, thereafter penetrating through the tract of Lissauer in the dorsal horn where they synapse with second-order neurons of nociceptive pathways. This disposition allows preferential interruption of pain pathway fibers while preserving larger-caliber myelinated fibers, which may play an inhibitory role, according to the classical “gate control” theory. In order to effectively achieve this, the lesion must be made in the ventrolateral side of the root and carried down to the deeper layer of the dorsal horn to destroy the second-order neuron of the spinothalamic pathways which might become hyperactive under deafferentation^6,7^ (Supplemental Fig. 5).

**2:32** Care must be taken to enter the DREZ on the ventrolateral side of the dorsolateral sulcus precisely at the entry of the dorsal rootlets, and to stay within the dorsal horn, in order not to damage the pyramidal tract laterally or the dorsal column medially.

**2:44** Performed on these principles, DREZ lesion achieves a well-defined territory of thermoalgic hypoesthesia limited to the metameric segments targeted and avoid late-onset neuropathic pain.

In this video, we demonstrate DREZ lesion at the cervical level for upper thoracic tumor (Pancoast-Tobias in this case) and lumbar DREZ lesion for tumor invading the lumbar plexus.

### Case 1

**3:06 Clinical Presentation.** The first case was a 55-year-old man with a slowly growing tumor in the right pulmonary apex invading the brachial plexus, which resulted in pain refractory to medical treatment including intrathecal morphine pump. Distribution of the pain was in the right C7, C8, and Th1 territories (Supplemental Fig. 6). Tumor involved the peripheral part of the cervical C7 root, but there was no compression of the spinal cord at the time of the surgery (Supplemental Fig. 7).

#### Approach

**3:38** DREZ lesioning was proposed targeting the C7, C8, and Th1 levels. Surgery was performed under general anesthesia and in the prone position. The approach was a C5–7 hemilaminectomy. In order to ensure sufficient exposure, we need to resect the entire hemilaminae from the articular processes laterally to deeply under the spinous process by rongeuring its base medially (Supplemental Fig. 8).

**3:54** After opening and suspending the dura and arachnoid covering the dorsolateral sulcus, the C7 to Th1—targets of the surgery—rootlets were exposed.

#### Step-by-Step Microsurgical DREZotomy

**4:00** The step-by-step procedure to perform DREZotomy is first shown at the level of the C7 root. After identification of the root, rootlets were everted to expose the dorsolateral sulcus. This may be rendered difficult at the cervical level by the lack of rootlet laxity. As was the case here, radiculomedullary arteries may be encountered, which could be dangerous to coagulate. On the other hand, at the dorsolateral sulcus, small pial vessels can be present; these should be coagulated to avoid untoward bleeding. The next step is incision of the pia mater on the ventrolateral side of the dorsolateral sulcus, precisely following its axis with a microknife of the ophthalmic type. In the case of cervical DREZ, the axis should be 35° in relation to the sagittal plane. Once the sulcus is incised, it should be opened so that lesioning with the bipolar coagulation forceps be performed under direct magnified vision. Microcoagulations were performed at 3–5 mm in depth in a dotted fashion every millimeter.

**4:57** For lesion making, the parameters of the bipolar machine are of importance and vary with the type of machine. It should be set on a delicate microsurgical mode with an initial power setting of 1 W. Coagulation is performed for 2–3 seconds at each point of coagulation carefully observing the tissue reaction in the open dorsolateral sulcus under the direct magnified vision of the operative microscope. A precise coagulation current is desirable for reproducibility, and testing and adjusting it on less consequential arachnoid is advisable.

As one can see, after this, the sulcus was largely opened, exposing the deeper gray to check the extent of the lesion.

**5:13** Observing these steps is crucial to achieve a good result. One must find the sulcus, open it, and coagulate within it at the appropriate depth and not encroaching the dorsal tract (cuneatus), medially, or the corticospinal tract, laterally.

**5:25** The same steps are shown at the lower portion of the same (C7) root, with eversion of the rootlets, incision of the pia mater, and then coagulation inside an open sulcus deep into the gray matter of the dorsal horn.

**5:35 Continuation of the DREZ at Other Levels.** The same process is repeated at the level of the C8 root, noting that as we transition toward the thoracic region the roots became more oblique and easier to maneuver, but the spinal cord has a smaller caliber, thus requiring to adapt the depth and angle of the lesion appropriately. In this particular case, the process was once more repeated at the level of Th1 root. At the end, final inspection showed a continuous lesion over the three targeted metameric levels with preservation of the medial region of the dorsal rootlets.

**6:23 Result and Postoperative Course.** Postoperatively the course was uneventful with hypoesthesia in the targeted area (Supplemental Fig. 9) and with pain subsiding for the rest of 1 year of life of the patient.

### Case 2

**6:31 Clinical Presentation.** The second case was a 61-year-old woman with a pseudomyxoma peritonei (Supplemental Fig. 10) with a slow local evolution. She presented with invasion of the right lumbosacral plexus and pain in the L2–5 territories (Supplemental Fig. 11) without any motor deficit, but bedridden due to the pain intensity. Given the territory involved, a L2–5 DREZotomy was decided.

**7:01 Approach.** A right hemilaminectomy from T11 to L1 was performed under general anesthesia and in the prone position. Care was taken to perform the hemilaminectomy as largely as possible in width, that is, from the articular processes laterally and including the base of the spinous process by rongeuring it (just as in the case presented of cervical DREZotomy).

#### Identification of Target Levels

**7:28** After opening and suspending the dura mater, the next step was arachnoid dissection to expose the conus and the lumbosacral roots. The next key step of the surgery was to identify the targeted roots. When performing DREZ at the lumbar and/or sacral level, this is essential since roots are closely packed and continuously disposed. Further, because of the limited laminectomy (in height), it is not possible to follow all of them from their point of entry into the spinal cord to their respective foramen. The landmark for the S1 root entry into the spinal cord is situated at 3 cm above the first coccygeal root.

**8:11** Muscle responses to electrical stimulation are essential for the identification of the other roots. In this case, responses in the quadratus lumborum muscles allowed identification of the L1 root and subsequently of the L2 DREZ level. Stimulation of motor roots was performed at a frequency of 2 Hz with a pulse with of 1000 μsec and an intensity of 200 μA (adjustable between 100 and 300 according to the response) using a bipolar stimulation probe (NIMBUS I Care stimulator) [Innopsys, www.innopsys.com].

Lesioning within the DREZ was then started just below L1 and carried all the way down to the DREZ above the S1 root.

**8:32 DREZ Lesioning.** Steps for lesioning are similar to those of cervical DREZotomy: exposure of the ventrolateral aspect of the dorsolateral sulcus in order to access the DREZ from the ventrolateral side. To do this, the roots were mobilized and everted toward the midline, which is easier than at the cervical level since the length of the roots gives slack. Next, small pial vessels were coagulated and incision of the sulcus was performed with an ophthalmic microknife to open it so as to perform the lesion under direct visual control. Note that an (important) artery running the dorsolateral sulcus was preserved. The lesion was performed with a bipolar forceps with a fine tip (0.2 mm), uninsulated over a 6-mm length, marked every millimeter to enable visual control of the depth of the lesion. Coagulations were performed every millimeter in a dotted fashion at a depth of 4 mm. The process was then repeated all along the targeted metameric segments in this case from L2 to L5 to cover the pain in those territories.

**9:48 Result and Postoperative Course.** Postoperative course was uneventful, with the expected hypoesthesia in the targeted territories resulting in a major decrease of pain (Supplemental Fig. 12). This led to a regain in function, the patient being able to resume walking with a crutch a few days after the surgery and freely at 6 weeks postoperatively. The effect was maintained at the 6-month follow-up visit.

### General Conclusion

**10:00** Results of DREZ lesioning for cancer pain patients have been reviewed^8,9^ recently by the senior author.^10^ Overall, more than three-quarters of the patients described a 75% decrease in pain with follow-ups ranging up to 48 months according to series (Supplemental Table 1).

**10:10** In conclusion, DREZ lesioning is recommended for well-selected patients suffering from cancer-related pain in the situation in which the lesion is well circumscribed, the territory of the pain well defined, and the general status and life expectancy compatible with an open neurosurgical procedure.

## Author Contributions

Primary surgeon: Brinzeu. Assistant surgeon: Mazzucchi. Editing and drafting the video and abstract: Brinzeu, Mazzucchi. Critically revising the work: Brinzeu, Mertens, Sindou. Reviewed submitted version of the work: Brinzeu, Mertens, Sindou. Approved the final version of the work on behalf of all authors: Brinzeu. Supervision: Sindou.

## Supplemental Information

### Online-Only Content

Supplemental material is available online.

*Supplemental Figures and Table*. https://thejns.org/doi/suppl/10.3171/2020.7.FOCVID2033.

## Supplemental Information

Supplemental Figures and Table
