# Supplementary material for: Microsurgical DREZ lesions for the control of cancer-related pain
Source: Neurosurg Focus Video. 2020 Oct 1;3(2):V14. doi: 10.3171/2020.7.FOCVID2033 (PMC9542504; doi:10.3171/2020.7.FOCVID2033)
Supplement: Supplemental Figures and Table [file SupplementalFiguresandTable_FOCVID20-33.pdf]

NEUROSURGICAL FOCUS VIDEO

ONLINE ONLY

Supplemental material

Microsurgical DREZ lesions for the control of cancer-related pain

Mazzucchi et al.

https://thejns.org/doi/abs/10.3171/2020.7.FOCVID2033

DISCLAIMER The *Journal of Neurosurgery* acknowledges that the following section is published verbatim as submitted by the authors and did not go through either the *Journal's* peer-review or editing process.

© 2020 The authors, CC BY 4.0 (http://creativecommons.org/licenses/by/4.0/)

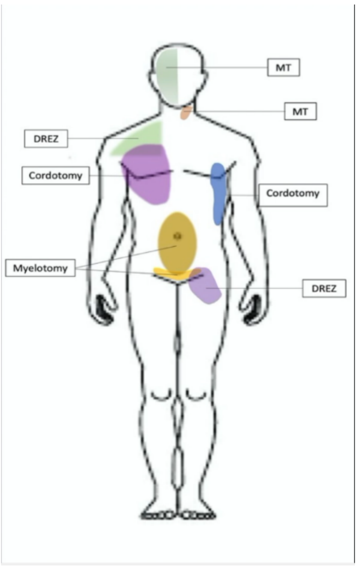

Supplemental Fig. 1: Localization of pain and relative surgical treatment. MT: mesencephalic tractotomy. DREZ: Dorsal Root Entry Zone

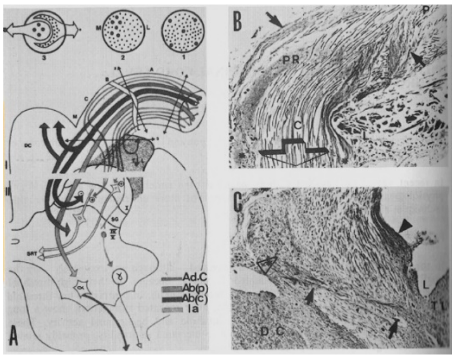

Supplemental Fig. 2: Organization of fibers at the posterior spinal cord-rootlet junction in Man. Image taken from the original article S*indou M, Quoex C, Baleydier C. Fiber organization at the posterior spinal cord-rootlet junction in man. The Journal of comparative neurology. Jan 1 1974;153(1):15-26*.

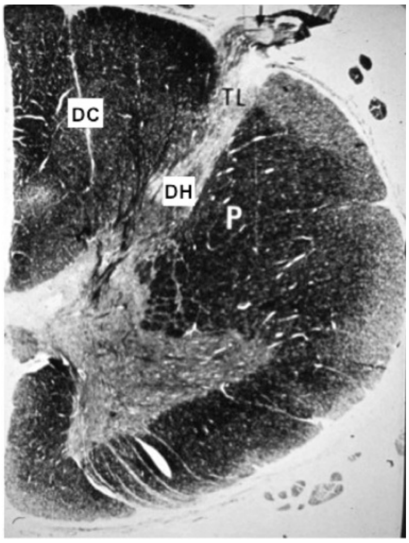

Supplemental Fig. 3: Spinal cord section, argentic coloration. DH: Dorsal Horn. DC: Dorsal Column. TL: Tract of Lissauer. P: Pyramidal tract.

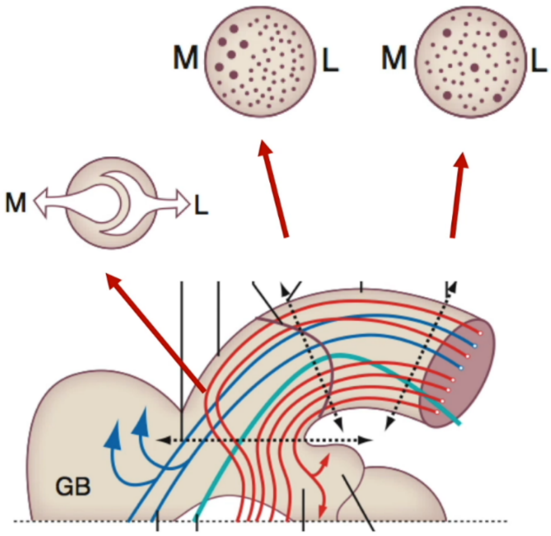

Supplemental Fig. 4: Schematic representation of the organization of large calibre myelinated fibres and smaller calibre myelinated and unmyelinated fibres at the transition between rootlet and spinal cord. M: Medial. L: Lateral. GB: Goll and Burdach fascicle.

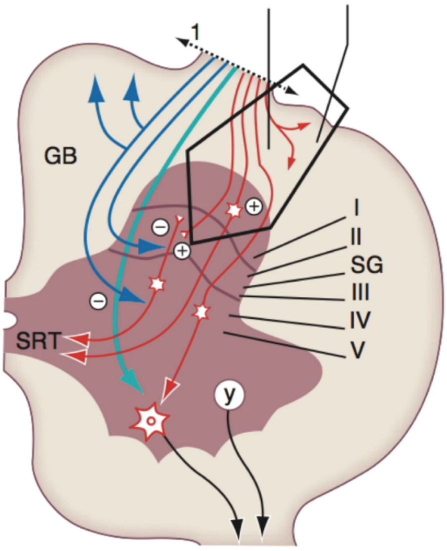

Supplemental Fig. 5: Picture of area involved in the dorsal root entry zone lesioning. The arrowhead represent the lesion performed during microsurgical DREZotomy. GB: Goll and Burdach fascicle. SRT: Spino-Reticulo-Thalamic tract. +: excitatory synapse. -: inhibitory synapse. I, II, III, IV, V: Rexed laminae. SG: Substantia Gelatinosa. Y: Gamma motor neuron.

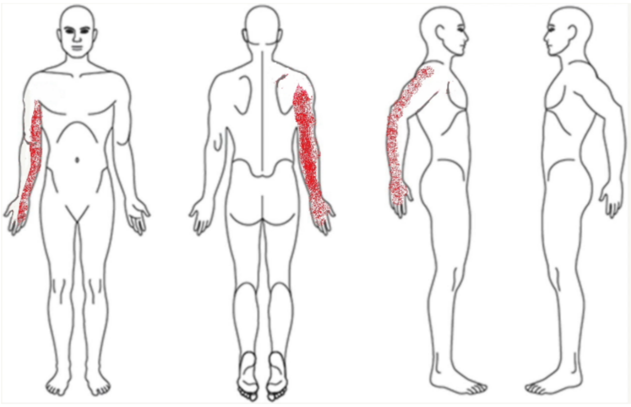

Supplemental Fig. 6: Pain map showing C7-T1 distribution of pain.

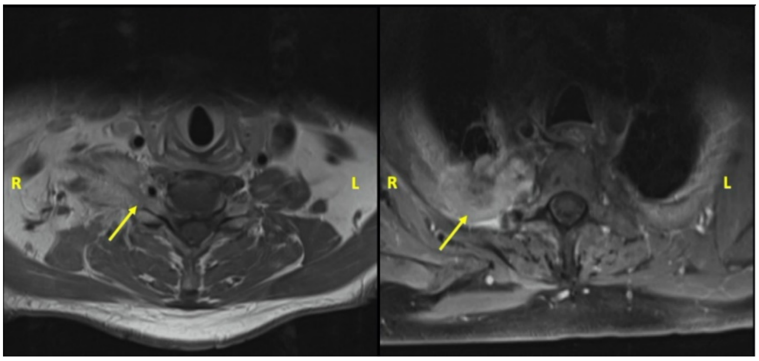

Supplemental Fig. 7: MRI scan images showing the pulmonary apex tumor invading cervical plexus.

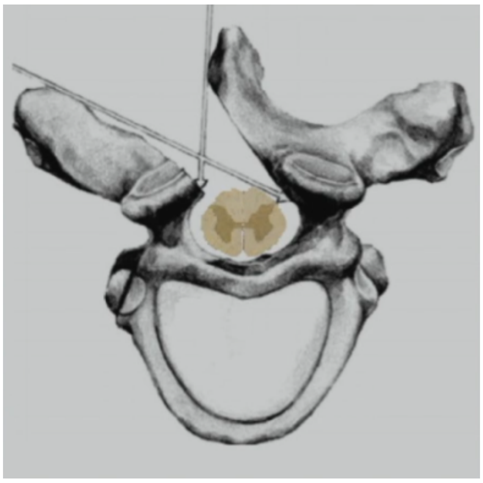

Supplemental Fig. 8: Schematic picture of the exposure obtained with hemilaminectomy.

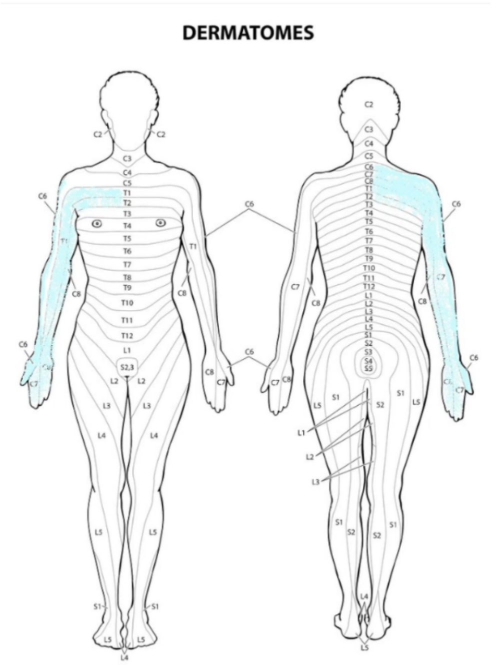

Supplemental Fig. 9: Distribution of post-operative hypoesthesia.

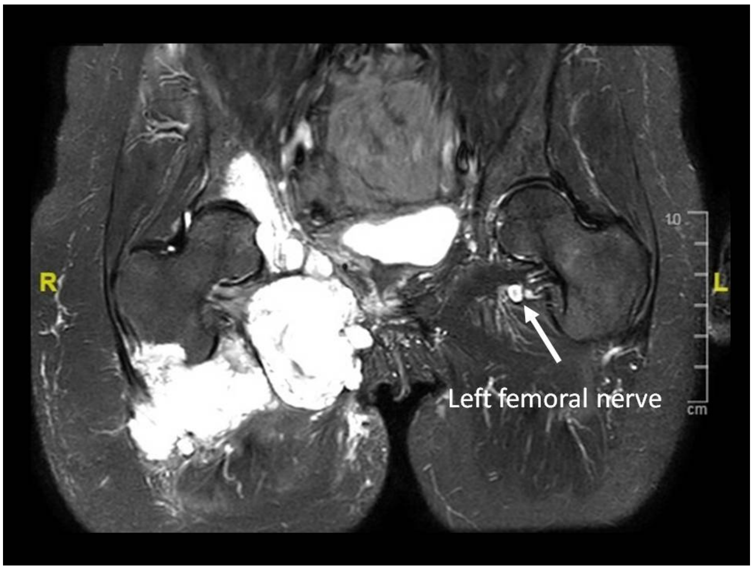

Supplemental Fig. 10: MRI coronal image showing the tumoral invasion of the right lumbosacral plexus.

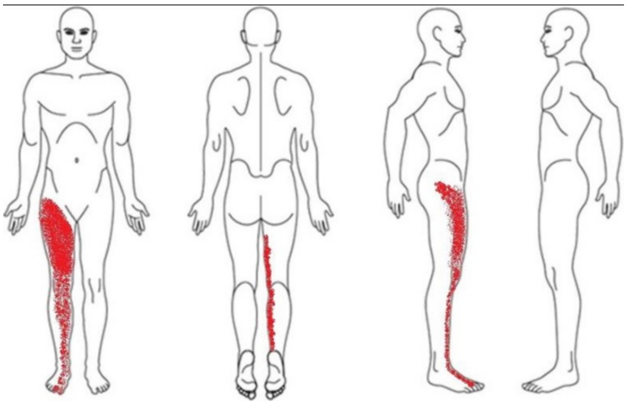

Supplemental Fig. 11: Pain distribution according to patient description and clinical assessment. The involved territory is comprised in dermatomes from L2 to L5.

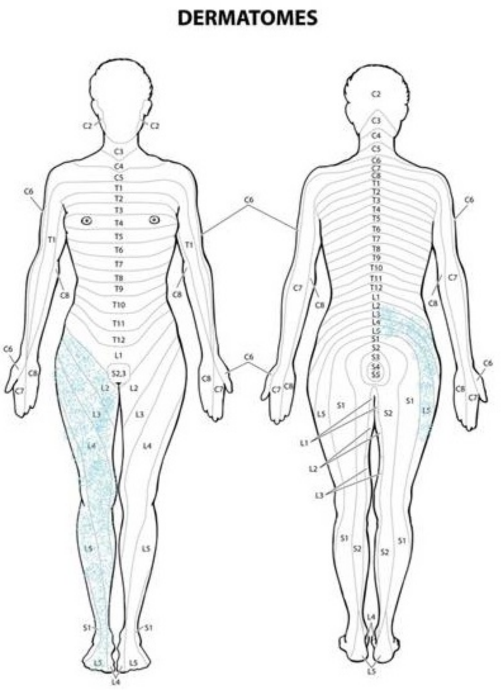

Supplemental Fig. 12: Representation of the area of analgesia observed in the post-operative period.

Reference	Technique	Number of patients	Follow-up, in months: range (mean)	Percentage of patients having > 75% relief
Nashold et al[6,80,81]	RF-Th	2 (cauda equina K)	8-4	100
Sindou and Lapras[39]	Microsurgery	13 (thoracic apex K)	1–30	90
Samii and Moringlane[82]	RF-Th	2 (breast K)	?	50
Powers et al[83]	Laser	3 (K)	?	100
Esposito et al[84]	Microsurgery	8 (K)	?	100
Kumagai et al[66]	RF-Th	1 (pelvic K)	2	0
Zeidman et al[85]	RF-Th	2 postradiation	29–48	100
Sindou	Microsurgery	46 (K): cervical MDT<br>35 (K): lumbar/sacral MDT	1–48	87<br>78
Rath et al[68]	RF-Th	2 postradiation	6–8	50
Teixeira et al[86]	RF-Th	7 postradiation	2–36	85
Ruiz-Juretschke et al[87]	RF-Th	3 (cervical K)	?	33
Kanpolat et al[72]	RF-Th	7 (K)	?	60
Taira (p.c.)	Microsurgery	3	> 1	?

Supplemental Table 1: Literature review of results of DREZ lesion in cancer pain. RF-Th: Radiofrequency-Thermocoagulation. MDT: Microsurgical DREZotomy. Adapted from: *Sindou M Dorsal Root Entry Zone Lesions in Burchiel K (ed) Surgical Management of Pain 576-592 Thieme Medical Publishers Inc New York 2014 ISBN-10 : 1604067519*
